# Enhancing the Thermal Conductivity of CNT/AlN/Silicone Rubber Composites by Using CNTs Directly Grown on AlN to Achieve a Reduced Filler Filling Ratio

**DOI:** 10.3390/nano14060528

**Published:** 2024-03-15

**Authors:** Naoyuki Matsumoto, Don N. Futaba, Takeo Yamada, Ken Kokubo

**Affiliations:** Nano Carbon Device Research Center, National Institute of Advanced Industrial Science and Technology (AIST), Tsukuba Central 5, 1-1-1 Higashi, Tsukuba 305-8565, Ibaraki, Japan

**Keywords:** carbon nanotubes, thermal conductivity, aluminum nitride, chemical vapor deposition, anti-hydrolysis, mechanical property

## Abstract

Achieving the thermal conductivity required for efficient heat management in semiconductors and other devices requires the integration of thermally conductive ceramic fillers at concentrations of 60 vol% or higher. However, an increased filler content often negatively affects the mechanical properties of the composite matrix, limiting its practical applicability. To address this issue, in this paper, we present a new strategy to reduce the required ceramic filler content: the use of a thermally conductive ceramic composite filler with carbon nanotubes (CNTs) grown on aluminum nitride (AlN). We combined catalyst coating technology with vacuum filtration to ensure that the catalyst was uniformly applied to micrometer-sized AlN particles, followed by the efficient and uniform synthesis of CNTs using a water-assisted process in a vertical furnace. By carefully controlling the number of vacuum filtration cycles and the growth time of the CNTs, we achieved precise control over the number and length of the CNT layers, thereby adjusting the properties of the composite to the intended specifications. When AlN/CNT hybrid fillers are incorporated into silicone rubber, while maintaining the mechanical properties of rubber, the thermal diffusivity achieved at reduced filler levels exceeds that of composites using AlN-only or simultaneous AlN and CNTs formulations. This demonstrates the critical influence of CNTs on AlN surfaces. Our study represents a significant advancement in the design of thermally conductive materials, with potential implications for a wide range of applications.

## 1. Introduction

The rise in the demand for compact electronic devices and high-power LED lighting systems has underscored the critical importance of efficient thermal management alongside advances in battery and device technology [1,2,3,4]. As a critical aspect of thermal management, the effective dissipation of heat generated by these devices to the surrounding environment has received considerable attention. The corresponding efforts include the use of various techniques, methods, and materials aimed at minimizing thermal resistance and utilizing materials with high thermal conductivity [4,5,6,7]. Among these approaches and tools, thermal interface materials (TIMs) are increasingly emerging as critical components that bridge the gap between heat sources (e.g., devices) and heat sinks (typically metal surfaces) [3,4,8]. Previous research has explored various avenues, including the integration of high-thermal-conductivity metals and alloys [5,9]; carbon-based materials such as graphene, graphene oxide (GO), and carbon nanotubes (CNTs) [10,11,12,13]; and ceramic fillers such as aluminum nitride (AlN), boron nitride (BN), silicon carbide (SiC), aluminum oxide (Al_2_O_3_), and zinc oxide (ZnO) [14,15,16,17,18,19,20,21] into silicone rubber or epoxy resin matrices. In addition, efforts have extended to the development of composite materials with malleable thermally conductive fillers capable of conforming to intricate device and heat sink geometries, thereby improving processability and heat dissipation efficiency [22,23].

AlN exhibits remarkable properties, including corrosion resistance, wear resistance, and exceptional thermal stability. Compared to materials such as Al_2_O_3_ (25–40 W/(m K)) [24] and silicon nitride (Si_3_N_4_, 180 W/(m K)) [25], AlN has a significantly higher isotropic thermal conductivity range (170–230 W/(m K), with a theoretical upper limit of 320 W/(m K)) [26]. This level is comparable with the isotopically high thermal conductivity of h-BN (in-plane: 200–280 W/(m K)) [27], a material characterized by its anisotropic thermal behavior. In addition, AlN has remarkable electrical resistivity (>1014 Ω cm) and a coefficient of thermal expansion similar to that of silicon semiconductors (4.3 × 10^−6^ K^−1^ at room temperature) and is resistant to the halogen gas plasma often used in semiconductor-manufacturing processes [28]. These properties make AlN a promising ceramic filler candidate for improving the thermal conductivity of resins and rubbers [14,29,30].

**Figure 1:**
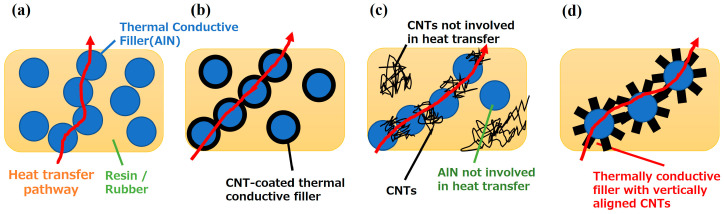
Concepts behind filler addition methods for CNT and AlN rubber/resin composites. (**a**) AlN, (**b**) CNT-coated AlN filler, (**c**) simultaneous addition of CNT + AlN, and (**d**) CNT/AlN composite filler. Red arrows: heat transfer pathway.

Despite the remarkable properties of AlN, its susceptibility to water-induced instability is a serious problem. When exposed to moisture, AlN oxidizes, denitrates, and hydrolyzes, forming aluminum hydroxide and corrosive ammonia. The result is a decrease in thermal conductivity, as the formation of non-thermal species takes precedence. Improving water resistance becomes an indispensable pursuit as we move toward the practical use of high-thermal-conductivity ceramic fillers such as AlN [31,32,33,34,35,36]. Generally, in order to effectively manage heat in semiconductors and similar devices, it is imperative to incorporate thermally conductive ceramic fillers at concentrations exceeding 60% by volume, thereby attaining the necessary thermal conductivity (Figure 1a). While increasing filler content increases thermal conductivity, it also affects the mechanical properties of TIM composites. Critical properties, such as tensile strength and elongation, are susceptible to decreasing [37,38]. In addition, increasing filler content affects the inherent flexibility of matrix resins or rubbers and subsequently compromises thermal dissipation efficiency by degrading adhesion to the contact surfaces of devices and heat sinks. Therefore, a critical challenge in the quest for superior thermal transfer properties remains strengthening the stability of AlN coupled with strategies to reduce the filler content in the rubber or resin matrix while maintaining high thermal conductivity.

Numerous efforts have focused on reducing the need for high AlN filler concentrations, including strategies such as particle coarsening and amalgamation with other fillers [39,40]. On the other hand, these methods, often resulting in research on improving AlN stability, particularly in terms of thwarting hydrolysis, have spawned diverse approaches. These include chemical treatments, such as surface conditioning and the use of surfactants, that modify surface chemistry [33]. In addition, shielding layers of materials such as GO, yttrium oxide (Y_2_O_3_), and silicon oxycarbonitride ceramics have been used to encapsulate AlN surfaces and insulate them from hydrolysis [34,35,36]. However, there is a potential problem: the inadvertent deposition of materials with inferior thermal conductivity compared to AlN could obstruct thermal conduction pathways, resulting in a decrease in overall thermal conductivity. In addition, the presence of organic substances, including surfactants, can induce coalescence in processes such as AlN powder molding and sintering [32]. It may also affect the stability and mechanical properties of rubber or resin compounds that interact with matrix components. Furthermore, coatings containing conductive substances, such as GO, could potentially affect the insulating properties of AlN [32]. While reports utilizing these strategies demonstrate increased water resistance, they do not report on the critical issue of reducing the filler content within the composite matrix.

CNTs, analogous to GO in their function of enhancing AlN stability (water resistance), have recently received considerable attention as potential fillers for high thermal conductivity composites, particularly TIMs. CNTs have a one-dimensional (1D) configuration, a high aspect ratio, and remarkable thermal conductivity (3000 W/(m K) for individual multiwall carbon nanotubes (MWCNTs) and 200 W/(m K) for bulk multi-walled CNTs at room temperature). With these properties, MWCNTs represent a promising high-thermal-conductivity filler, similar to AlN [41,42,43,44]. While pure silicone rubber typically has low thermal conductivity (0.1–0.3 W/(m K)), both its corrosion resistance and thermal conductivity significantly improve when it is filled with high-thermal-conductivity ceramic powders [24]. This improvement in properties expands its range of applications. Its blending with CNTs has demonstrated increased thermal conductivity and reduced the required filler content in composites, including those with ceramic fillers such as AlN and CNTs [20,41]. However, the simultaneous integration of ceramics, such as AlN and CNTs, can be complicated. In addition, the difficulty of AlN stabilization (water resistance) persists, and the presence of non-contributing CNTs within thermal pathways can potentially trigger a decline in mechanical properties due to the need for increased filler content (Figure 1c).

We have previously reported the successful synthesis of vertically aligned CNTs on flat AlN substrates using water-assisted chemical vapor deposition (CVD), i.e., the “super-growth method” [45]. The commonly used method of the vapor-phase deposition of an Fe catalyst is not suitable for the deposition of spherical particles, as shown in Figure 1. In this paper, we present a new strategy for reducing the filler content of AlN and improving the water resistance of AlN: CNT/AlN fillers, in which CNTs are grown on AlN particles used as a thermal conductive filler, as shown in Figure 1d. Specifically, we have focused on the application of Fe catalyst coating methods to both AlN filler particles and CNT synthesis processes. In this pursuit, we used wet-based catalyst coating techniques, in particular dip coating and vacuum filtration coating, to coat AlN particles with an Fe catalyst. We then proceeded to vertically grow CNTs directly on the AlN particles, a step aimed at improving the water resistance of AlN. In addition, we demonstrate the exceptional properties of this CNT/AlN composite as a thermally conductive filler in silicone rubber matrices. By creating efficient thermal conduction pathways within the matrix, as shown in Figure 1d, we increased thermal conductivity while ensuring reduced filler content. For this purpose, we directly mixed CNT/AlN particles with silicone rubber. In the process of conducting this study, we successfully exercised precise control over the structural properties (length and number of layers) of the synthesized CNTs. This was made possible by meticulously adjusting the catalyst coating conditions applied to the AlN particles and regulating the CNT synthesis duration through a straightforward methodology. Moreover, by integrating the synthesized CNT/AlN filler with silicone rubber, we achieved about twice the thermal conductivity of AlN alone at about half the filler addition rate while maintaining the mechanical properties of rubber. This was evident when compared with previous reports involving other strategies for producing AlN-based composites either in isolation or in conjunction with CNTs.

## 2. Materials and Methods

### 2.1. Coating of Fe Catalyst onto AlN Particles

**Figure 2:**
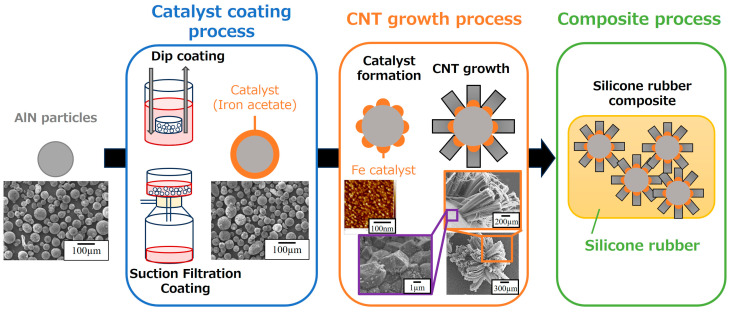
Scheme for the preparation of CNT/AlN composite filler and CNT/AlN/Silicone rubber composite. CNT growth process inset shows SEM and AFM images of Fe particles on an AlN substrate after partial exfoliation of CNTs grown over the entire particle after CNT synthesis, as well as SEM images showing vertical CNT growth from the AlN substrate.

AlN particles selected for this study had a particle size distribution characterized by D_50_ = 80 µm and D_90_ = 122 µm (HFS-80, Tokuyama Corporation, Tokyo, Japan). Prior to use, these AlN particles were ultrasonically cleaned using acetone and ethanol for 5 min, followed by vacuum drying at 100 °C. The dried AlN particles were then immersed in a 30 mM (CH_3_COO)_2_Fe/ethanol solution. A vacuum pump and filter were used for the vacuum-filtration-coating procedure. This setup facilitated the controlled filtration of the iron acetate solution onto the surfaces of the AlN particles, resulting in the deposition of iron acetate (Fe catalyst) onto the AlN substrates. To achieve the desired amount of iron acetate deposition, both the deposition and vacuum filtration steps were repeated iteratively, ranging from 1 to 5 cycles. In the dip-coating process, the prepared dried AlN particles were enclosed in a stainless-steel mesh container. This container had an aperture size of 0.20 mm, a wire diameter of 0.53 mm, and an open porosity of 62.6%. The AlN-loaded container was then immersed in a 30 mM (CH_3_COO)_2_Fe/ethanol solution. The container was pulled out of the solution at a controlled rate of 0.5 mm/s, and this immersion/pulling sequence was repeated three times, as shown in the catalyst-coating process in Figure 2.

### 2.2. Synthesis of Vertically Aligned CNTs on Fe/AlN Particles

AlN particles coated with iron acetate (Fe catalyst) via both vacuum filtration and dip-coating methods were introduced into a mesh dish with an aperture size of 0.35 mm, a wire diameter of 0.21 mm, and an open porosity of 29.9%. This mesh dish was then placed in a horizontal 1-inch quartz tube furnace. The temperature of the furnace was ramped up to 750 °C at a controlled rate of 50 °C/min. The furnace was then held at this temperature for an additional 10 min while maintaining an atmosphere of H_2_:N_2_ at a ratio of 9:1. This particular environment was created to facilitate the formation of Fe catalyst nanoparticles. Next, the synthesis of CNTs was performed using water-assisted chemical vapor deposition (CVD). A mixture consisting of a carbon feedstock of C_2_H_2_ (0.5%), a growth enhancer of H_2_O at a concentration of about 250 ppm, and a N_2_ carrier gas was introduced into the furnace. This introduction was carried out over a period of 0–20 min while maintaining the temperature at 750 °C. This process, referred to in Figure 2 as the CNT growth process, allowed the growth of vertically aligned CNTs [46].

### 2.3. Preparation of CNT/AlN/Silicone Rubber Nanocomposites

CNT/AlN/silicone rubber nanocomposites were prepared in accordance with a standardized protocol [47]. First, CNT/AlN particles were dispersed directly into a silicone precursor (KE-106 silicone rubber base compound, Shin-Etsu Chemical Co., Ltd., Tokyo, Japan) via overnight stirring at 50 rpm. A curing agent (CAT-RG, Shin-Etsu Chemical Co., Ltd., Tokyo, Japan) was then added to the CNT/silicone precursor mixture at a weight ratio of 10% with respect to the silicone precursor. The resulting mixture was homogenized and degassed using a planetary centrifugal vacuum mixer (ARV-310, THINKY Co., Ltd., Laguna Hills, CA, USA). To fabricate the CNT/AlN/silicone rubber nanocomposites, the mixture was hardened in a heated mold under a pressure of approximately 10 MPa at 150 °C for 30 min (Figure 2, Composite Process). This process resulted in the formation of the CNT/AlN/silicone rubber nanocomposites.

### 2.4. Characterizations

The color difference between Fe/AlN particles subjected to suction filtration and dip coating was evaluated using a colorimeter/whiteness meter (NW-12, Nippon Denshoku Industries CO., Ltd., Tokyo, Japan) with a sample size (*n*) of 10. The Fe and Al proportions were quantified using a fluorescence X-ray instrument (EDXL 300, Rigaku Corporation, Tokyo, Japan), with *n* = 5. Both digital microscopy (VHX-5000, Keyence, Osaka, Japan) and scanning electron microscopy (SEM; VE-9800, Keyence) were used to investigate the general morphology of CNTs on AlN particles. The determination of the CNT length measured from the AlN surface to the CNT tip via the SEM images was performed using using the SEM’s built-in high-resolution dimensional measurement function. A minimum of 20 samples were taken for each sample. Quantification of the CNT layers was performed using transmission electron microscopy (TEM; EM-002B, TOPCON, Tokyo, Japan). For TEM observations, CNT-ethanol dispersions were deposited on Cu TEM grids, and the number of layers was measured over more than 50 CNTs. The thermal diffusivity (through plane) of the CNT/AlN/rubber composite was evaluated at room temperature under ambient conditions using a flash analyzer (LFA-464, NETZSCH, Selb, Germany) equipped with a Xenon lamp. To visualize the thermal conduction pathways (CNT/AlN) within the silicone rubber, the rubber was immersed in chloroform. Dissolution of the silicone rubber was carried out via stirring at high speed (500 rpm) overnight using a high-speed shaker (ASCM-1, High-Speed Shaker, AS ONE Corporation, Osaka, Japan). After dissolution, the resulting sample (CNT/AlN) was observed using SEM.

## 3. Results and Discussion

### 3.1. Coating of Fe Catalyst on AlN Particles

**Figure 3:**
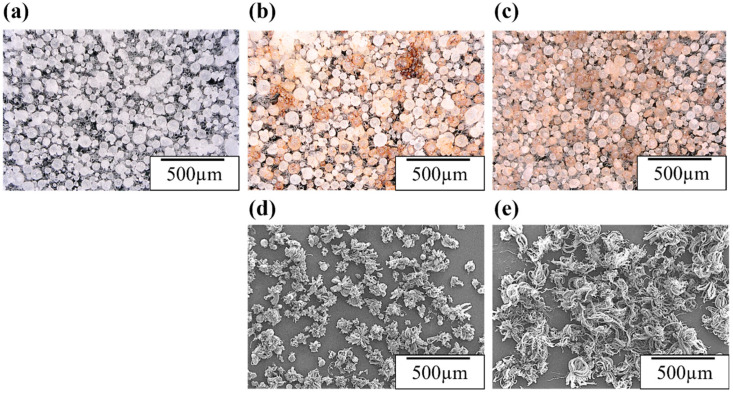
Overall view of AlN particles coated with Fe catalyst source (iron acetate) using different coating methods: (**a**) AlN particles (before coating), (**b**) dip coating, and (**c**) suction filtration coating. SEM images of CNT/AlN composite filler after the synthesis of CNTs directly on Fe/AlN particles prepared using different coating methods: (**d**) dip coating; (**e**) suction filtration coating.

First, we investigated how the uniformity of the Fe catalyst on AlN particles changes with different catalyst-coating methods. In particular, we found that Fe acetate as a catalyst source could be more uniformly distributed on the AlN particle surface via suction filtration compared to dip coating. The uniform distribution of Fe acetate was confirmed through color difference measurements. When Fe (II) acetate was applied to AlN, the sample surface changed to a reddish-brown color. This color change was quantified in the L*a*b* color space, a color model commonly used to represent object colors. In the Lab color space, both a* and b* values become positive: L* represents lightness, a* represents the green-to-red direction, and b* represents the blue-to-yellow direction [48]. As the values increase, the color becomes more vivid, while values near the center (where a* and b* are both zero) indicate more muted colors. In essence, a thicker (higher amount of) Fe acetate (the Fe catalyst) coating on AlN results in increased a* and b* values, resulting in decreased brightness (L*). Digital microscope images of the AlN particles before being coated with the Fe catalyst and of AlN particles coated with Fe acetate (Fe catalyst) via suction filtration or dip coating (100 °C, 1 h, vacuum-dried) are shown in Figure 3a–c. In addition, Table 1 shows the mean and standard deviation of color differences for the AlN particles and Fe/AlN particles measured at 10 points for each condition. From the visual inspection of Figure 3 and the comparison of color differences in Table 1, it is evident that the use of suction filtration to apply a 30 mM Fe acetate solution resulted in a more uniform distribution of Fe acetate compared to dip coating. When comparing the color differences, the dip coating (a* = 3.5 ± 1.9, b* = 18.9 ± 3.4, L* = 82.8 ± 4.2) showed color differences similar to the suction filtration coating (applied five times) (a* = 3.9 ± 1.1, b* = 15.6 ± 1.9, L* = 77.4 ± 2.8) but with approximately half the standard deviation. This quantitative assessment by color difference confirms the uniform distribution of Fe acetate (Fe catalyst) not only visually but also quantitatively. Similar results were obtained from the semi-quantitative values of Al and Fe, as well as the standard deviation of the Al/Fe ratio obtained from X-ray fluorescence (XRF) measurements, as shown in Table S1. In addition, the comparison of coating repetition (three vs. five times) in Table 1 shows that repeated application of the same 30 mM solution via suction filtration led to an increase in color differences: a*—1.6 to 3.9, b*—10.3 to 15.6, and L*—79.3 to 77.4. This shift indicates a darker red-brown color attributed to Fe acetate (Fe catalyst). This result illustrates how the amount of Fe acetate on AlN particles (Fe catalyst film thickness) can be controlled by adjusting the number of suction filtration rounds, demonstrating the potential of this simple technique for controlling the amount of Fe acetate through filtration repetitions.

Next, we demonstrate the controlled and uniform synthesis of CNTs on AlN particles coated with an Fe catalyst using either the suction filtration or dip-coating methods. Figure 3d,e show SEM images of CNTs synthesized via the CVD method on Fe acetate/AlN substrates prepared via suction filtration or dip coating, respectively. When the Fe catalyst was applied using suction filtration, the CNTs grew uniformly, while dip coating resulted in non-uniform CNT growth, with some particles showing no CNT growth at all. As shown in the AFM and SEM insets of the growth process in Figure 2, the CNTs grew vertically from the Fe catalyst particles on the AlN substrate. The CNT lengths were determined from the SEM images (corresponding to the distance from the AlN particle surface to the CNT tip) in Figure 3d,e, confirming that the CNT lengths for suction filtration and dip coating were 350 ± 52 μm and 368 ± 97 μm, respectively. The smaller error in CNT length for suction filtration further supports the uniform growth of CNTs. Although the resultant CNT growth was highly dependent on the synthesis conditions, it was also strongly dependent on the thickness and uniformity of the Fe catalyst coating as well as the support layer (such as Al_2_O_3_) [49]. Essentially, under synthesis conditions where the carbon source gas (such as ethylene) is uniformly fed to the Fe catalyst on AlN particles, optimal CNT growth conditions vary with Fe catalyst thickness. Consequently, the non-uniform CNT growth observed in the dip-coated specimens (as shown in Figure 3d) suggests that the non-uniform thickness of the Fe acetate catalyst layer is a contributing factor. This notion is consistent with the results regarding the uniformity of the Fe catalyst coating as indicated by the color difference data shown in Figure 3d,e and Table 1.

### 3.2. Control of CNT Growth on AlN Particles

**Figure 4:**
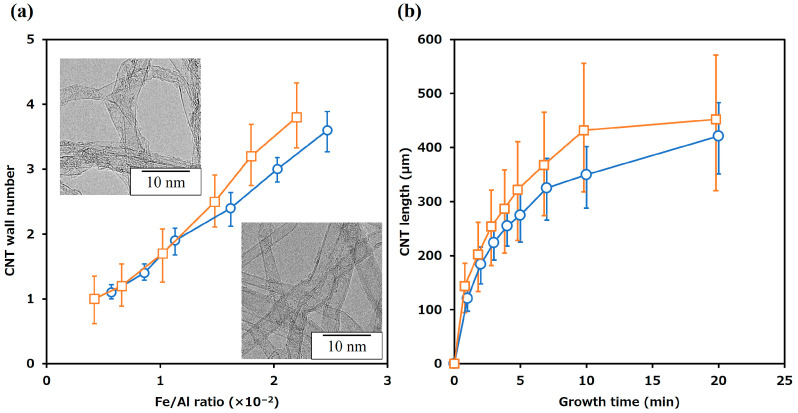
Structural changes of CNTs synthesized on AlN with varying amounts of Fe catalyst and synthesis time: (**a**) catalyst amount and number of CNT layers; (**b**) synthesis time and CNT length. Squares (orange lines): dip coating; circles (blue lines): vacuum filtration coating.

We further investigated the influence of the catalyst-coating methods, namely, suction filtration and dip coating, on the growth of CNTs on Fe/AlN particles. The number of CNT layers was determined from TEM images (*n* = 50, as shown in the inset of Figure 4a) of CNTs synthesized on Fe/AlN particles coated using both methods. The relationship between the Fe-to-Al ratio on AlN and the number of CNT layers was established and shown in Figure 4a. The Fe-to-Al ratio was calculated based on the Fe and Al content (%) obtained via the X-ray fluorescence analysis of Fe/AlN particles, and the average value of ten measurements was considered to be the Fe/Al ratio. In addition, the adjustment of the Fe acetate (Fe catalyst) content in the dip-coating process was controlled by modifying the number of dip cycles, similar to the suction filtration process: an increase in the number of dips resulted in a higher Fe acetate content. Regardless of the catalyst-coating method employed, an increase in the Fe/Al ratio corresponded to an increase in the number of CNT layers synthesized. However, when comparing the error bars on each plot showing the layer number distribution, it becomes evident that the layer number distribution was narrower for suction filtration synthesis compared to that for dip coating. We clarified the relationship between the thickness (deposition amount) of the Fe catalyst and the number of CNT layers. An increase in the thickness of the Fe catalyst resulted in an increase in the number of CNT layers (diameter). This trend is consistent with previous research indicating that the observed increase in number of layers of the CNTs with the increased Fe thickness likely results from the increase in the average catalyst nanoparticle size, which has been reported previously [49].

Therefore, the wider distribution of layer counts observed in dip coating can be attributed to the lower uniformity of catalyst thickness, while suction filtration, which allows for uniform catalyst application, showed a narrower layer count distribution. Figure 4b illustrates the relationship between CNT length (from the AlN particle surface to the CNT tip) and synthesis time. The CNT length was measured via SEM images using the measurement software provided with the scanning electron microscope, and measurements were taken for 30 CNT/AlN particles to calculate the average length and deviation. Figure 4b shows that regardless of the coating method, the CNT length increased with longer synthesis times. These growth processes are consistent with previous findings and confirm that the CNT length on AlN particles can be controlled by adjusting the synthesis time [50].

With these results, we have demonstrated the feasibility of controlling the number of layers (diameter) and length of CNTs grown on AlN substrates using the simple adjustment methods of Fe catalyst coating, either by varying the number of dips in dip coating or the synthesis time. Furthermore, we found that the ability of suction filtration to uniformly coat the Fe catalyst leads to uniform CNT growth, which is different from dip coating. These results are important, as the number of CNT layers and CNT length are two factors that determine electrical and thermal properties. We realize that a number of other factors, such as straightness and the absence of crystalline defects, were within the scope of our synthetic control. We found that as the number of layers increased, the thermal conductivity of the CNTs improved. Further, we found that CNTs with 2–3 layers had the highest electrical conductivity [51]. On the other hand, CNT length affects not only electrical and thermal properties but also mechanical properties. Longer CNTs lower the percolation threshold and improve electrical conductivity and tensile strength when used to fabricate CNT films [52,53]. The process presented in this paper, which facilitates the control of CNT structures (regarding number of layers and length) in a straightforward manner, is well suited for synthesizing CNTs with tailored properties and creating composites with application-specific material properties.

### 3.3. Thermal Properties of CNT/AlN/Slicon Rubber Composites

**Figure 5:**
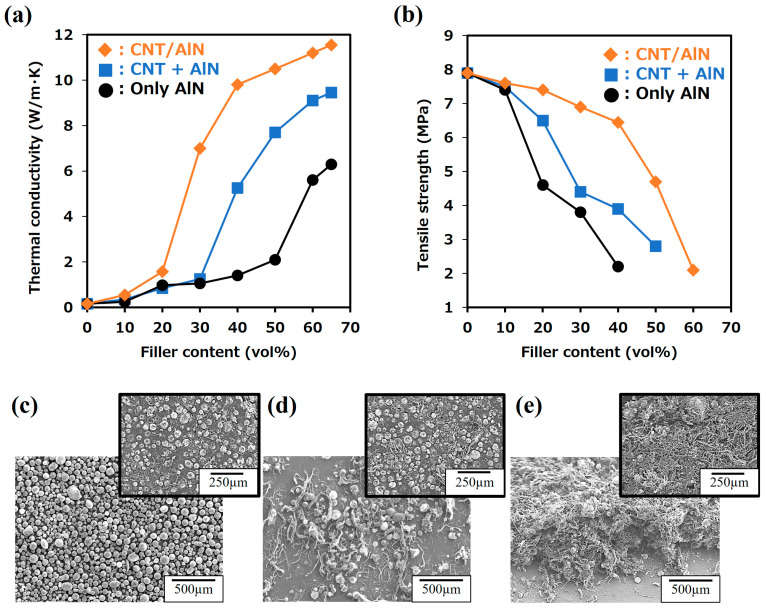
Variation in (**a**) thermal conductivity and (**b**) tensile strength with filler content in silicone rubber composites with AlN filler, AlN + CNT filler addition, and CNT/AlN composite filler. SEM images of CNT and AlN filler network after removal of silicone rubber: (**c**) AlN filler, (**d**) AlN + CNT filler addition, and (**e**) CNT/AlN composite filler (inset: SEM images of the surface of each composite). The filler content of AlN, AlN + CNT, and CNT/AlN in each composite was 65, 60, and 50 vol%, respectively.

Using the suction filtration method to uniformly coat the Fe catalyst and enable uniform growth of CNTs on AlN particles (CNT/AlN composite fillers), we fabricated composites by combining these fillers with silicone rubber (CNT/AlN/silicone rubber composites). We have demonstrated that these composites exhibit superior thermal properties at a lower filler content than that of the AlN alone and of AlN with CNTs (AlN + CNT). Specifically, we fabricated CNT/AlN composite fillers and silicon rubber composites and examined the relationship between thermal conductivity and filler content. Figure 5a shows the thermal conductivity of composites consisting of AlN only and with the simultaneous addition of AlN and CNTs and of CNT/AlN composite fillers synthesized via the suction filtration method for Fe catalyst deposition on AlN added to silicon rubber (CNT/AlN/silicon rubber composites). The preparation conditions of the CNT/AlN composite fillers used in Figure 5a were adjusted so that they were structurally similar to CNTs (ZEONANO^®^ SG101, Tokyo, Japan) when added simultaneously with AlN. The filtration cycle in suction filtration was set to three (Fe/Al ratio: 0.0066) using Fe/AlN particles with an average layer number of 1.2 and an average length of 350 µm (10 min synthesis time). With the incorporation of CNT/AlN composite fillers, the onset of thermal conductivity increased for composites containing AlN only or both AlN and CNTs and occurred at relatively high filler proportions (50 vol% and 30 vol%, respectively). In contrast, the CNT/AlN composite exhibited an exceptionally early-onset filling level at ~20 vol%. In addition, the filler content required to achieve ~20 W/mK occurs at 30 vol%, ~45 vol%, and >60 vol% for CNT/AlN, AlN + CNT, and AlN, respectively. Furthermore, the maximum observed thermal conductivity occurred for the CNT/AlN composite and at a relatively low filler content. This result demonstrates the importance of incorporating the CNTs and AlN into the polymer matrix together rather than as separate components.

The use of CNT/AlN composite fillers facilitates the formation of thermal conduction paths via the bonding of CNTs to AlN particles, resulting in a lower minimum filler content (percolation threshold) required to exhibit thermal conductivity, as shown in the conceptual diagram in Figure 1. In addition, by using CNT/AlN composite fillers, the non-contributing CNTs and AlN in terms of thermal conductivity are minimized by the composite formation of CNTs and AlN compared to adding the same quantities of CNTs and AlN simultaneously. Thus, a further reduction in filler content can be achieved compared to the simultaneous addition of AlN and CNTs. Furthermore, Figure 5a shows that the thermal conductivity of CNT/AlN composites was 10.5 W/m K (filler content: 50 vol%), while composites containing only AlN or CNTs or both AlN and CNT exhibited thermal conductivities of 6.3 W/m K (filler content: 65 vol%) and 9.1 W/m K (filler content: 60 vol%), respectively, indicating high thermal conductivity regardless of filler content. Figure 5b also shows the tensile strength of these composites. When AlN alone or AlN + CNT alone were combined with silicone rubber, the tensile strength increased with the addition of AlN alone or CNT alone, but in both cases, the tensile strength decreased significantly at additions above 20 vol%. On the other hand, in the case of CNT/AlN filler addition, the tensile strength remained almost unchanged up to 40 vol%: the tensile strength decreased above 40 vol%. These results indicate that the CNT/AlN composite filler not only reduces the amount of filler added to about half of that of AlN alone while maintaining the mechanical properties (tensile strength) but also improves thermal conductivity. In addition, the simultaneous incorporation of AlN and CNTs, which can be easily integrated into composites, improves thermal conductivity and mechanical properties compared to AlN alone. Therefore, the choice between CNT and AlN or the simultaneous introduction of CNTs and AlN should be based on the thermal conductivity, mechanical properties, and cost considerations relevant to the target application.

To elucidate the mechanism of enabling high thermal conductivity at a low filler content while maintaining mechanical strength, we tried to observe the filler network (AlN and CNT) in each composite, as shown in Figure 5c–e. Although we tried to freeze-slice these composites using a cryomicrotome, it was difficult to prevent the CNTs from becoming detached during sectioning. Therefore, surface observation (SEM) and a technique involving the dissolution of the base material (silicone rubber) with chloroform were used to observe each composite. In the case of the addition of AlN alone, shown in Figure 5c, contact between AlN particles was observed. When AlN and CNTs were added simultaneously (Figure 5d), contact between AlN particles and the bridging of CNTs with AlN particles were observed, along with the presence of individual AlN and CNTs that did not depend on heat transfer. Figure 5e confirms the three-dimensional interconnection of CNTs grown on AlN with other CNTs within the composite structure. From these observations, as shown schematically in Figure 1, it was concluded that the simultaneous addition of AlN and CNTs results in a lower filler content and higher thermal conductivity due to the contact between AlN and CNTs and the bridging between them compared to the addition of AlN. In contrast, when a CNT/AlN composite filler is used, the direct growth of CNTs on AlN suppresses the thermal conductivity of the non-participating CNTs and AlN (Figure 1d). In addition, the formation of a three-dimensional thermal conduction network between the fillers (Figure 5e) enables efficient heat transfer, resulting in the development of thermal conductivity at lower filler additions compared to that for the simultaneous addition of CNTs and AlN, as shown in Figure 5a. This phenomenon highlights the important role of 3D CNT networks in promoting thermal conduction within composites and, in particular, suggests that networks formed by CNTs synthesized directly on AlN are an important factor in improving thermal properties even at low filler concentrations.

On the other hand, the CNTs were uniformly dispersed and properly aligned within the matrix, as shown in Figure 5e; they interacted with the matrix to increase material resistance to mechanical stress. First, the three-dimensional network of CNTs on AlN effectively disperses mechanical (tensile) stress from external sources, reducing stress concentrations [53]. As a result, the overall strength of the material is increased, and the occurrence of cracks and fractures is prevented. In addition, when CNTs are uniformly dispersed and aligned within the matrix, the interactions between individual CNTs are optimized, maximizing the tensile strength of the material [53]. Furthermore, the right density of CNTs promotes good adhesion to the matrix, ensuring that tensile loads are evenly distributed. As a result of these interactions, the material can withstand higher tensile stresses, resulting in improved durability. This is provided by the uniform distribution and correct density of the CNTs within the material. Therefore, CNT/matrix composites show potential as materials with high tensile strength and excellent durability network.

## 4. Conclusions

This study provides evidence of the utility of vertically oriented CNT/AlN composite fillers on thermally conductive ceramic fillers, namely, AlN particles. In addition, uniform synthesis of CNTs on AlN surfaces was achieved by using the wet catalyst coating method of suction filtration. By adjusting the filtration cycle of the iron catalyst source (iron acetate), it was possible to control the number of layers and the length of the CNTs, thus controlling the growth time of the CNTs. In addition, because this process is performed at room temperature and atmospheric pressure, the fabrication time of CNT/AlN composite fillers, from AlN catalyst deposition to CNT growth, is remarkably short (within 1 h). This fast and convenient process is a significant advantage. The composite of the fabricated CNT/AlN fillers with silicone rubber showed that even with reduced filler content, superior thermal conductivity can be achieved compared to that of conventional AlN alone or the simultaneous addition of AlN and CNT. CNT synthesis on the AlN surface also contributes to the reduction in odor (ammonia odor) resulting from the reaction between AlN and moisture, as shown in Figure S1. These results suggest that CNTs encapsulating AlN and Fe particles may also help prevent direct contact between the Fe catalyst and the silicone rubber, thereby mitigating rubber degradation. Therefore, when these CNT/AlN composite fillers are incorporated into resins or rubbers as thermal conductivity enhancers for TIM materials, they are expected to provide high thermal conductivity without compromising the mechanical properties of the matrix material, even at reduced filler content. However, it should be noted that the CNT/AlN/silicon rubber composite presented in this study exhibits conductivity on the order of several mS/m due to the synthesis of CNTs on AlN surfaces. For applications requiring thermal materials with insulating properties, such as semiconductors, ensuring insulation is often imperative. Recent studies have reported the wrapping of CNTs with insulating BN [54], and the adoption of these insulation technologies for CNT/AlN composites is expected to be pursued in the future. In this study, we have not only introduced the concept of novel fillers vertically oriented CNTs on the surface of thermally conductive materials but also demonstrated their ability to efficiently create three-dimensional heat conduction paths, thereby enhancing material properties. This concept is expected to have a ripple effect on materials such as CNT composites, which exhibit excellent electrical properties. In addition to the demonstrated improvements in thermal conductivity and mechanical properties, the developed CNT/AlN/silicone rubber composites hold promise for various practical applications, including TIMs, electronic packaging, and thermal management systems.

## Figures and Tables

**Table 1:** Change in color difference of AlN beads due to iron acetate application.

		a*		b*		L*	
		Value	STD	Value	STD	Value	STD
AlN Beads		0.03	0.01	4.0	0.3	95.0	2.2
Vacuum filtration	3 times	1.6	0.85	10.3	1.6	79.3	2.4
	5 times	3.9	1.1	15.6	1.9	77.4	2.8
Dip coating		3.5	1.9	18.9	3.4	82.8	4.2

## Data Availability

Data are contained within the article.

## Supplementary Materials

The following supporting information can be downloaded at https://www.mdpi.com/article/10.3390/nano14060528/s1. Table S1: Amount of Fe catalyst on AlN and its standard deviation in X-ray fluorescence (XRF) measurement; Figure S1: Variation in ammonia concentration in AlN or AlN/CNT composite filler as a function of time of atmospheric exposure (atmospheric exposure conditions: Temperature: 24.3 °C, Humidity: 75%).
