# Supplementary material for: Enhancing the Thermal Conductivity of CNT/AlN/Silicone Rubber Composites by Using CNTs Directly Grown on AlN to Achieve a Reduced Filler Filling Ratio
Source: Nanomaterials (Basel). 2024 Mar 15;14(6):528. doi: 10.3390/nano14060528 (PMC10974482; doi:10.3390/nano14060528)
Supplement: Supplementary file 1 [file nanomaterials-14-00528-s001.zip › nanomaterials-2913773-supplementary.pdf]

nanomaterials

MDPI

Supplementary Materials

Enhancing the Thermal Conductivity of CNT/AlN/Silicone Rubber Composites by Using CNTs Directly Grown on AlN to Achieve a Reduced Filler Filling Ratio

Naoyuki Matsumoto *, Don N. Futaba, Takeo Yamada and Ken Kokubo

Nano Carbon Device Research Center, National Institute of Advanced Industrial Science and Technology (AIST), Tsukuba Central 5, 1-1-1 Higashi, Tsukuba 305-8565, Ibaraki, Japan

* Correspondence: matsumoto-naoyuki@aist.go.jp; Tel.: +81-29-861-4551

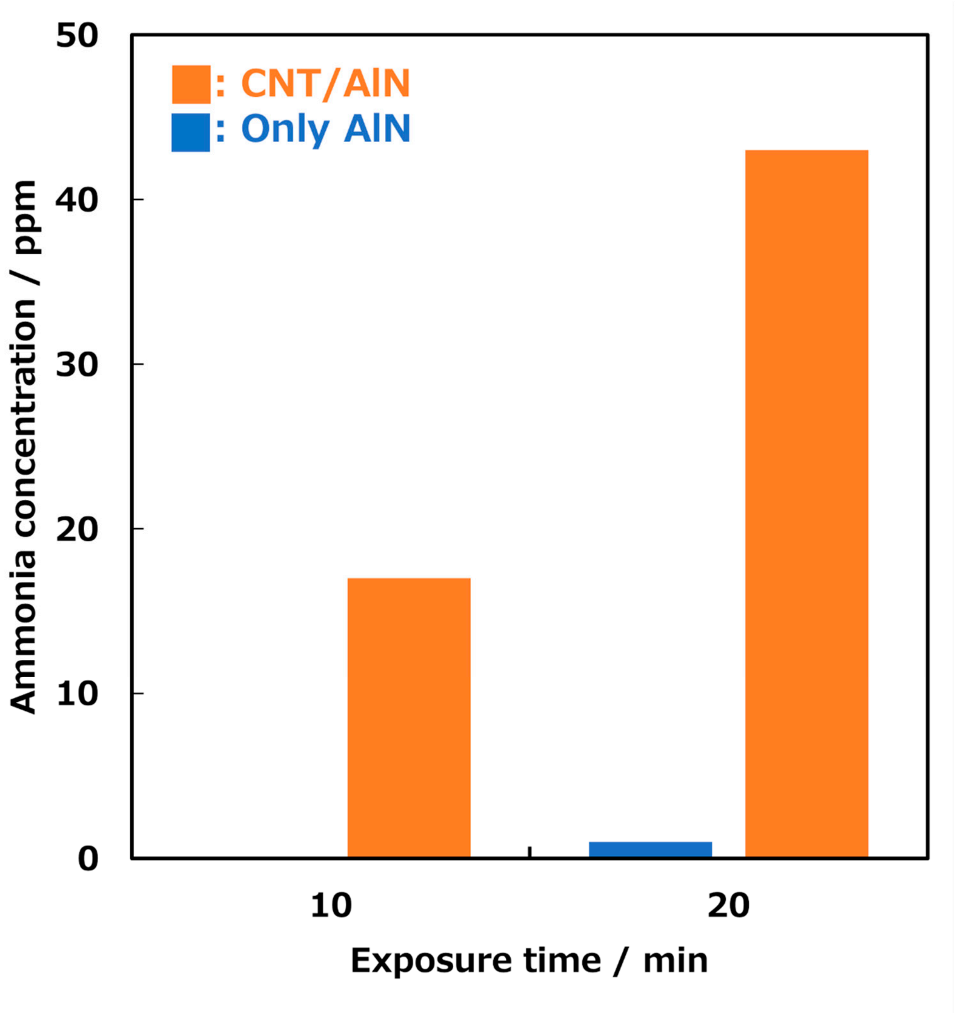

Figure S1. Variation in ammonia concentration in AlN or AlN/CNT composite filler as a function of time of atmospheric exposure (atmospheric exposure conditions: Temperature: 24.3 °C, Humidity: 75%).

Table S1. Amount of Fe catalyst on AlN and its standard deviation in X-ray fluorescence (XRF) measurement.

	Fe content ($\times10^{-2}$ mass%)	Al content (mass%)	Fe/Al ratio	
			Value ($\times10^{-3}$)	STD ($\times10^{-5}$)
Vacuum filtration	2.42	10.6	2.28	2.80
Dip coating	2.51	12.4	2.03	9.82
